# Diet and Meal Pattern Determinants of Glucose Levels and Variability in Adults with and without Prediabetes or Early-Onset Type 2 Diabetes: A Pilot Study

**DOI:** 10.3390/nu16091295

**Published:** 2024-04-26

**Authors:** Leinys S. Santos-Báez, Diana A. Díaz-Rizzolo, Collin J. Popp, Delaney Shaw, Keenan S. Fine, Annemarie Altomare, Marie-Pierre St-Onge, Emily N. C. Manoogian, Satchidananda Panda, Bin Cheng, Blandine Laferrère

**Affiliations:** 1 Division of Endocrinology, Nutrition Obesity Research Center, Columbia University Irving Medical Center, New York, NY 10032, USA; 2 Health Science Faculty, Universitat Oberta de Catalunya (UOC), 08018 Barcelona, Spain; 3 Institute for Excellence in Health Equity, Department of Population Health, New York Langone Health Grossman School of Medicine, New York, NY 10016, USA; 4 Center of Excellence for Sleep & Circadian Research, Division of General Medicine, Department of Medicine, Columbia University Irving Medical Center, New York, NY 10032, USA; 5 Regulatory Biology Laboratory, Salk Institute for Biological Studies, La Jolla, CA 92037, USA; emanoogian@salk.edu (E.N.C.M.);; 6 Department of Biostatistics, Columbia University Irving Medical Center, New York, NY 10032, USA

**Keywords:** continuous glucose monitoring, diet composition, meal timing, normoglycemic, dysglycemic

## Abstract

This observational pilot study examined the association between diet, meal pattern and glucose over a 2-week period under free-living conditions in 26 adults with dysglycemia (D-GLYC) and 14 with normoglycemia (N-GLYC). We hypothesized that a prolonged eating window and late eating occasions (EOs), along with a higher dietary carbohydrate intake, would result in higher glucose levels and glucose variability (GV). General linear models were run with meal timing with time-stamped photographs in real time, and diet composition by dietary recalls, and their variability (SD), as predictors and glucose variables (mean glucose, mean amplitude of glucose excursions [MAGE], largest amplitude of glucose excursions [LAGE] and GV) as dependent variables. After adjusting for calories and nutrients, a later eating midpoint predicted a lower GV (β = −2.3, SE = 1.0, *p* = 0.03) in D-GLYC, while a later last EO predicted a higher GV (β = 1.5, SE = 0.6, *p* = 0.04) in N-GLYC. A higher carbohydrate intake predicted a higher MAGE (β = 0.9, SE = 0.4, *p* = 0.02) and GV (β = 0.4, SE = 0.2, *p* = 0.04) in N-GLYC, but not D-GLYC. In summary, our data suggest that meal patterns interact with dietary composition and should be evaluated as potential modifiable determinants of glucose in adults with and without dysglycemia. Future research should evaluate causality with controlled diets.

## 1. Introduction

Dietary carbohydrates (CHOs) are recognized as major determinants of glucose levels and glucose variability (GV) [1,2]. Interventional and epidemiological studies suggest that an excess calorie intake [3], particularly food with a high glycemic load [4,5], are key contributors to overall obesity risk and metabolic dysfunction [6,7]. In addition, meal timing is an important component of peripheral clocks that influence robust circadian rhythms; therefore, the frequency and timing of eating occasions (EOs) and the duration of the eating window are gaining traction as important risk factors for metabolic outcomes [8,9,10,11].

Higher GV is associated with the development and worsening of type 2 diabetes (T2D) [12]. Although CHO content and quality have been shown to predict GV in adults with and without dysglycemia [13], there is a paucity of data on the influence of meal timing and dietary behaviors on daily glucose excursions in individuals with prediabetes or normoglycemia. Moreover, it is yet unclear whether meal timing and/or its variability can impact daily glycemic fluctuations. The use of continuous glucose monitoring systems (CGMs) combined with dietary monitoring in real time with smartphone apps [14,15,16] provides a unique opportunity to assess the impact of meal timing and diet and their variability on mean glucose levels and glucose excursions with minimal daily life disruptions [14,17].

In this pilot study, we aim to investigate the association between meal patterns and dietary intake with glucose monitored using a CGM in free-living conditions in individuals with and without dysglycemia. We hypothesize that a prolonged eating window and late EO times would predict greater glucose levels and glucose variability (GV), and that dietary composition would be the main driver of glucose levels and GV. Additionally, we hypothesize that these effects would be more pronounced in individuals with dysglycemia.

## 2. Methods

### 2.1. Study Design

Data for this pilot study were derived from two separate studies conducted at the Columbia University Irving Medical Center: the New York Time Restricted EATing to improve cardiometabolic health (NY-TREAT) randomized clinical trial [18] and the Glucose, Activity, Diet, and Sleep Assessment Study (GLADS), a cross-sectional observational study. All participants signed informed consent prior to enrollment. Data on glucose, meal patterns and diet were collected during a 2-week assessment period under free-living conditions (Supplemental Figure S1). Data from the NY-TREAT study were collected during a 2-week ambulatory period under free-living conditions prior to randomization.

### 2.2. Participants

Inclusion and exclusion criteria for both groups are listed in Supplemental Table S1. In brief, the 26 D-GLYC participants were between the ages of 50 and 75 years, with overweight or obesity (body mass index [BMI] 25–44.9 kg/m^2^) and prediabetes or T2D treated with diet and/or metformin and HbA1C < 7.5%, with a habitual long daily eating window (≥14 h). The 14 participants in N-GLYC were ≥18 years, had a BMI of 20–35 kg/m^2^ and no history of prediabetes or T2D. For both studies, exclusion criteria included a history of sleep disorders, shift work, bariatric surgery and current engagement in weight loss with or without medication.

### 2.3. Blood Pressure and Anthropometry

Blood pressure was measured in triplicate with a digital monitor (Digital Automatic Blood Pressure Monitor HEM-907XL, Omron Healthcare, Inc., Kyoto, Japan) in a standardized manner after a 10 min rest in a sitting position. The first reading was discarded and the average of the second and third readings was recorded. Height and weight were measured in duplicate. In N-GLYC, height was measured to the nearest 1 mm using a mechanical stadiometer (SECA 222, Seca, Hamburg, Germany) and weight was measured after voiding, in light clothing, to the nearest 0.1 kg using a calibrated digital scale (Tanita BWB-800, Tanita, Arlington Heights, IL, USA). In D-GLYC, participants were asked to void, remove all garments and jewelry, and were provided a hospital gown by the study staff, after which triplicate anthropometric measures were obtained; body weight was measured to the nearest 0.1 kg (Ohaus Champ General Purpose Bench Scale, Ohaus Corp., Pine Brook, NJ, USA); height was measured to the nearest 1 mm using a stadiometer (Holtain Ltd., Crymych, UK), as previously described [18]. The BMI was calculated as body weight in kilograms (kg) divided by body height in meters squared. All anthropometric measures were obtained on day 1 of the 2-week assessment.

### 2.4. Glucose

Interstitial glucose was recorded every 15 min with a CGM (Abbott Freestyle Libre Pro, Abbott Park, IL, USA) [19] placed on the non-dominant upper arm during the first visit (Supplemental Figure S1). At the end of the 2 weeks, CGM data were downloaded from LibreView software Version 1.0 and were subject to data review. The following data were removed from analyses: (1) readings obtained the day of CGM insertion until 4:00 a.m. the following day to accommodate for equilibrium; (2) data of the entire 24 h day when a CGM dislodgement event or malfunction occurred, with dislodgement events reported by the participant and accompanied by missing data with or without a preceding malfunction (i.e., at least eight consecutive glucose readings below 50 mg/dL immediately before dislodgement). For N-GLYC, glucose data were used from day 2 to 14. In D-GLYC, only data from days 2 to 12 were included, as the diet was controlled on days 13 and 14 [18]. After a data review, a total of 17,082 individual glucose values were available from N-GLYC participants, of which 15,980 (93.5%) were analyzed, while 29,807 glucose readings were available from D-GLYC participants, of which 25,021 (83.9%) were analyzed.

Data generated with the CGM were computed with EasyGV v8.6 software [20] and included: (1) mean glucose; (2) mean amplitude of glycemic excursion (MAGE), ignoring excursions of 1 standard deviation (SD) or less; (3) the largest amplitude of glycemic excursion (LAGE), calculated as the difference between the maximum and minimum blood glucose values; and (4) SD of glucose as a marker of GV.

### 2.5. Dietary Intake

Participants were instructed to complete up to six 24 h dietary recalls using the validated web-based Automated Self-Administered 24-hour^®^ (ASA24^®^) Dietary Assessment Tool [21] on non-consecutive weekdays and at least one weekend day with instructions to maintain their typical dietary intake. Participants received e-mail reminders from the study staff to ensure data collection. Time-stamped dietary recalls were validated with a photo-based smartphone application, as described below. Responses from dietary recalls were coded and downloaded directly from the ASA24^®^ backend, which included parameters of dietary intake, including caloric intake, grams of CHO, protein, total fat, sugar, fiber and alcohol. The percentage of daily CHO, protein, total fat and the sugar-to-CHO ratio and fiber-to-CHO ratio were calculated for each participant, and the within-person variability of dietary intake and percentages were calculated as the SD of the 2-week energy and nutrient intake.

### 2.6. Eating Patterns

On day 1, participants downloaded the validated research software application myCircadianClock Version 7.5.5 (mCC app, Salk Institute, La Jolla, CA, USA) on their personal smartphone and received a 10 min coaching session by the study staff on how to record time-stamped photos, in real time, of all food and caloric beverages immediately prior to ingestion [16,22]. The study staff had access to the backend of the app and provided feedback and assistance as needed. All data were transferred to a remote server immediately upon submission [16]. Given the interval of a 24 h day from wake to sleep extending past midnight for some participants, the 24 h period was arbitrarily adjusted to start at 4:00 a.m. and end at 3:59 a.m. the following day to account for EOs occurring after 12:00 a.m. EOs were defined as any caloric intake that takes place throughout a 24 h day except water.

Eating pattern parameters assessed included the 2-week mean eating window (interval during which 95% of EOs occur) [23], as well as the 2-week mean ± SD of daily number of EOs, time of first and time of last EO and eating midpoint (median timepoint between the first and last EO). Adherence to logging was defined as (1) the documentation of two or more EOs separated by 5 h and (2) logging for at least 70% of the days. To ensure adherence, the research staff tracked all participant entries and sent push notifications directly through the mCC application. Participants also received automatic push reminders to log their EOs, including beverages.

### 2.7. Statistical Analyses

All data were screened for outliers. Categorical variables, such as sex, age, ethnicity, race and prediabetes or T2D status, were compared with a chi-squared test (Table 1). Continuous variables were first tested for normal distribution using the Shapiro–Wilk test. Pearson’s and Spearman’s correlations were performed to assess relationships in parametric/nonparametric variables. Independent *t* tests and Mann–Whitney U tests were used to compare continuous variables in parametric and nonparametric data, respectively. Continuous variables were represented as mean ± SD for each group.

Cross-sectional continuous data were analyzed using a general linear model to examine the associations between diet, eating pattern, behavioral variability and glucose parameters. Eating pattern parameters were analyzed with adjustments for daily dietary composition, including caloric intake and grams of CHO, protein, total fat, sugar, fiber and alcohol consumed.

Baseline characteristics were explored descriptively for all participants using SPSS (IBM SPSS Statistics for Windows, Version 29.0 IBM Corp, Armonk, NY, USA). General linear model analyses were carried out using statistical software package SAS version 9.4 (Cary, NC, USA) and data were expressed as mean ± SE. The statistical significance level was set at α = 0.05. All analyses were performed first with both groups combined and then in each group separately.

## 3. Results

A total of 43 participants (14 N-GLYC and 29 D-GLYC) completed the 2-week assessment. CGM malfunctions occurred for three participants in D-GLYC who were removed from the analyses (Supplemental Figure S2); therefore, 40 participants (n = 14 N-GLYC and n = 26 D-GLYC) were include in the final analysis.

### 3.1. Participant Characteristics

Participants were predominantly women in both groups; D-GLYC participants were older (*p* < 0.001), had a higher diastolic blood pressure (*p* = 0.004), weight (*p* = 0.004) and BMI (*p* < 0.001), and 23 of them had prediabetes (*p* < 0.001) (Table 1 and Table 2) compared to N-GLYC. Body composition measurements of fat mass and fat-free mass were available for the D-GLYC group, but there were no significant associations between body composition and glucose parameters (Supplemental Table S2).

Adherence with meal logging was excellent, with over 90% of days displaying good logging in both groups (Table 2), and although groups differed in the duration of ambulatory period, there were no differences in eating pattern parameters (daily number of EOs, eating window, time of first EO, time of last EO and eating midpoint). There was an average of 5 ± 1 dietary recalls in each group, with no group differences in the mean calorie intake (*p* = 0.571) despite a greater alcohol intake in N-GLYC (*p* = 0.008), and a trend for a greater percentage of energy intake from fat in D-GLYC (*p* = 0.051) (Table 2).

**Figure 1:**
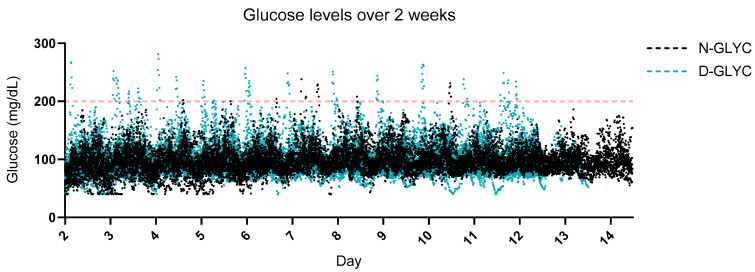
Glucose excursions over a 2-week period. 2-week continuous glucose monitoring (CGM) daily glucose measurements synchronized across the two groups. Glucose parameters did not differ significantly between groups (Table 2). Red dashed line = indicates the threshold for interstitial glucose readings above 200 mg/dL.

Although N-GLYC participants had a smaller range of glucose readings over 2 weeks (Figure 1), the mean glucose, MAGE, LAGE and GV were not different between groups (Table 2). In D-GLYC only, the mean glycosylated hemoglobin (HbA1c) was 5.9 ± 0.3, while the homeostatic model assessment for insulin resistance (HOMA-IR) was 4.9 ± 4.1 (Table 2). Correlations between HOMA-IR and glucose parameters were not significant (Supplemental Table S3), and there was a significant negative correlation between HOMA-IR and fiber intake, fiber intake variability and alcohol intake (Supplemental Table S4).

### 3.2. Effect of Meal Pattern and Its Variability on Mean Glucose, Glucose Excursions and GV

We assessed whether meal patterns influenced glucose and its variability. Meal patterns did not predict glucose levels nor GV in the combined analyses. In N-GLYC, a later time of last EO predicted a higher GV (β = 1.5, SE = 0.6, *p* = 0.04). In D-GLYC, a higher variability in the number of EOs predicted a higher mean glucose (β = 6.6, SE = 2.4, *p* = 0.02), while a later eating midpoint was associated with a lower GV (β = −2.3, SE = 0.1, *p* = 0.03).

### 3.3. Effect of Diet Composition and Its Variability on Mean Glucose, Glucose Excursions and GV

**Figure 2:**
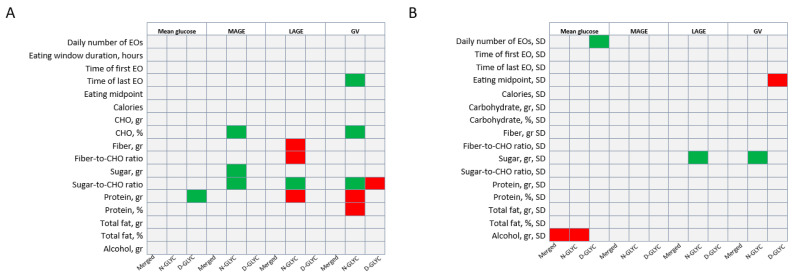
Diet and eating pattern (**A**) and their variability (**B**) as predictors of glucose parameters in the entire sample (merged) and subgroup analyses. Summary of behavioral predictors of glucose parameters. Eating patterns were adjusted for caloric intake, carbohydrate (gr), protein (gr), total fat (gr), sugar (gr), fiber (gr) and alcohol (gr). (**A**) Diet and eating pattern behavior over two weeks as predictors of mean glucose and glucose variability. (**B**) Diet and eating pattern behavior variability over two weeks as predictors of mean glucose and glucose variability. Green boxes indicate significant positive predictions; red boxes indicate significantly negative predictions. Data analyzed using a generalized linear model. Significance set at 0.05. Abbreviations: CHO—carbohydrate; EO—eating occasion; GV—glucose variability defined as glucose SD; LAGE—largest amplitude of glucose excursion; MAGE—mean amplitude of glucose excursion; SD—standard deviation.

We then assessed whether diet composition influenced glucose and its variability. Contrary to our hypothesis, neither dietary CHO, sugar nor fiber predicted mean glucose in combined or separate analyses by cohort. On the other hand, a higher protein intake predicted higher mean glucose only in D-GLYC (β = 0.2, SE = 0.1, *p* = 0.03). Alcohol intake variability negatively predicted mean glucose in the combined analyses (β = −0.4, SE = 0.2, *p* = 0.03) and N-GLYC (β = −0.7, SE = 0.3, *p* = 0.04), but not in D-GLYC (Figure 2).

No element of diet composition predicted MAGE or LAGE in the combined analyses or in the D-GLYC. In N-GLYC only, higher intakes of CHO (β = 0.9, SE = 0.4, *p* = 0.02), sugar (β = 0.3, SE = 0.1, *p* = 0.02) and sugar-to-CHO ratio (β = 75.8, SE = 27.7, *p* = 0.02) were associated with a higher MAGE, a higher sugar-to-CHO ratio (β = 166.6, SE = 60.7, *p* = 0.02) and higher sugar variability (β = 1.0, SE = 0.3, *p* = 0.01) were associated with a higher LAGE; and a higher fiber (β = −1.7, SE = 0.8, *p* = 0.04), fiber-to-CHO ratio (β = −490.2, SE = 224.6, *p* = 0.05) and protein intake (β = −0.7, SE = 0.2 = 0.01) was associated with lower LAGE (Figure 2).

Finally, none of the dietary parameters or their variability predicted GV in the combined analyses. However, in the N-GLYC only, a higher percentage of energy from CHO intake (β = 0.4, SE = 0.2, *p* = 0.03) and a higher sugar-to-CHO ratio (β = 35.8, SE = 11.5, *p* = 0.01) were associated with a higher GV, while a higher protein intake in grams (β = −0.1, SE = 0.04, *p* = 0.03) and percentage of energy (β = −0.7, SE = 0.3, *p* = 0.04) were associated with a lower GV. Counterintuitively, in the D-GLYC group, a higher sugar-to-CHO ratio was associated with a lower GV (β = −0.4, SE = 0.2, *p* = 0.03) (Figure 2).

## 4. Discussion

In this study, we investigated the effect of eating patterns, diet composition and the variability of these behaviors on glucose levels and GV in adults with and without dysglycemia assessed over a 2-week period under free-living conditions. There were no clear differences in glucose control between the two groups, despite one group being classified as having dysglycemia and being older. This finding could be related to the D-GLYC group having a mean glycosylated hemoglobin (HbA1c) of 5.9 ± 0.3%, putting this group’s average value on the lower range of prediabetes classification [24].

Our data supported our hypothesis that some aspects of eating patterns could modulate glucose levels and GV in segregated analyses. In D-GLYC, a higher variability of daily EOs was associated with a higher mean glucose, which supported our hypothesis, as irregular meal intakes have been shown to be associated with a higher insulin resistance [25]. Our data also revealed that a later time of the last EO was associated with a higher GV in N-GLYC, while a later eating midpoint was associated with a lower GV in D-GLYC. Our results for meal timing seemed to replicate previous findings in N-GLYC, but not D-GLYC. A long eating window and later timing have all been linked to glucose intolerance and higher glucose levels [26] and an earlier eating window was shown to enhance metabolic benefits of TRE in individuals at risk of T2D [27], with evidence from randomized clinical trials indicating that following a time-restricted eating diet (TRE) [27,28,29] leads to improvements in glucose homeostasis. However, midpoint results are best assessed in the context of first and last EO timing, and the effect of diet seemed to surpass that of the eating window. While the reasons underlying group discrepancies in response to meal frequency and timing remain unclear, it is plausible that the observed differences reflected the stringent inclusion criteria employed in the D-GLYC group. Notably, participation in this group necessitated a prolonged eating window. Future studies employing larger and more heterogeneous participant pools, encompassing a wider range of eating window durations, are warranted to address this question. Additionally, implementing controlled dietary interventions with manipulated eating window durations could offer further insights into the potential role of meal timing.

As expected, dietary CHO contributed to glucose excursions. Interestingly, this was only observed in the N-GLYC group. These findings also contradicted prior findings with CHO intake being one of the primary drivers of glucose levels and fluctuations in adults with and without T2D [1,2,30,31,32,33]. To further explore this finding, we segregated CHO intake by type. A higher sugar and a sugar-to-CHO ratio predicted higher glucose excursions and greater GV in N-GLYC, but a higher sugar-to-CHO ratio was associated with lower GV in D-GLYC. Sugar intake is a recognized determinant of the increased prevalence of T2D [34], and previous reports revealed that, compared to a habitual diet, a healthy low-CHO intake promoted greater reductions in fasting plasma glucose and a higher percentage of time in range glucose readings [35], with the effects of sugars on the blood glucose response being proportional to the CHO they replaced [36,37]. Fiber intake and the fiber-to-CHO ratio were associated with lower glucose excursions in N-GLYC, but no associations were found in D-GLYC. Fiber is widely favored as a dietary complement for glucose management, as it reduces post-prandial glucose excursions [38,39]; however, this favorable effect is only apparent when at least 25 g of daily fiber is consumed [40], and our study showed a lower fiber intake than the current recommendations in both groups, which may have dampened any potential protecting effect of fiber reported in the literature. Our results replicated the known effects of sugar and fiber on glucose found in the literature in the N-GLYC group only; however, the inverse relationship was found between the sugar-to-CHO ratio and GV, and the lack of associations seen between fiber and glucose levels in D-GLYC suggests that the recommended amount of sugar and fiber intake may be of greater importance in high-risk groups.

A higher protein intake was associated with lower glucose excursions and GV in N-GLYC, but with a higher 2-week mean glucose in D-GLYC. These findings partially aligned with prior research, as a higher protein intake has been linked to an improved GV and post-prandial glucose response, lower risk of developing T2D and higher likelihood of T2D remission [41,42,43]. Although protein findings were present in a normoglycemic cohort, the counterintuitive observation between protein and mean glucose in D-GLYC may also be attributed to a lack of substantial CHO restrictions, as a high protein intake does not affect plasma glucose in people with T2D unless caloric and CHO intake are markedly reduced [44].

Participants in the N-GLYC group were younger and reported a higher alcohol consumption, which aligned with research indicating a gradual decline in alcohol intake with age [45]. Our study showed a significant association between higher alcohol intake variability and higher mean glucose for N-GLYC, supporting the well-established connections between a high alcohol intake and elevated sugar content [46]. While alcohol consumption in the D-GLYC group was highly variable, it did not predict daily glucose levels or variability. This observation could indicate a moderate drinking pattern, which is generally recognized to have minimal or even a slight reduction in impact on blood glucose levels in individuals with T2D [47].

The strengths of this study included the ecological assessment of diet and meal pattern under free-living conditions in younger and older adults of racially and ethnically diverse backgrounds with varying glucose tolerance levels. This allowed for the exploration of differences in behavior and their variability as predictors of glucose, glucose excursions and variability. To our knowledge, we were the first to assess the relationships between diet, meal pattern and their variability and 24 h glucose parameters in normoglycemic adults and adults with mild dysglycemia; hence, this study paves the way for considering a comprehensive dietary behavior approach of CGM data interpretation.

There were limitations that warranted acknowledgement. The cross-sectional and observational study design prevented establishing causality, age discrepancies between groups may have confounded the results and the small sample size reduced analytical power, potentially yielding chance findings. Some findings may have resulted from selection bias given the rigid inclusion criteria in both studies (e.g., eating window); however, differences in anthropometric parameters were present between groups, making results generalizable to a wider population. Unmeasured factors (e.g., differences in diet during morning, midday or evening) could have acted as confounders, and reported intake was not evaluated for plausibility and could have led to participant recall bias. Nevertheless, the ASA24 is the most appropriate method to assess diet in free-living conditions, and participants underwent extensive training for ASA24 and had repeated measures, as previously suggested, to strengthen the data generated [48]. The study would have further been strengthened by incorporating comprehensive assessments of body composition, including the proportion of fat mass and fat-free mass in both groups, as these are known to impact glucose metabolism [49,50]. Additionally, lower post-prandial glucose concentrations have been shown to correlate with hunger rating and the subsequent energy intake [51]; therefore, qualitative assessments of hunger and post-prandial glucose dips could have provided valuable insights and helped elucidate the drivers of meal timing and diet behaviors and could be added in future studies.

## 5. Conclusions

In summary, these pilot data suggested that there were different modifiable dietary and eating pattern determinants that may have impacted the 24 h glucose patterns and their day-to-day variability in adults with and without dysglycemia. Eating frequency predicted mean glucose in D-GLYC, while the time of the last EO predicted GV in N-GLYC, and the eating midpoint predicted GV in D-GLYC. For diet, CHO, sugar and fiber intake predicted glucose excursions and GV in N-GLYC, but not in D-GLYC. These results underscore the need to further evaluate the role of meal frequency, timing and regularity as concomitant dietary pattern predictors in healthy and metabolically impaired adults. Future research should evaluate causality with controlled diets.

## Figures and Tables

**Table 1:** Participant characteristics.

Variable		N-GLYC	D-GLYC	Pearson Chi-Sq
		n (%)	n (%)	
Gender	Male	5 (35.7)	7 (26.9)	0.563
	Female	9 (64.3)	19 (73.1)	
Age group	<35 years	4 (28.6)	0 (0)	**<0.001**
	35–44 years	3 (21.4)	0 (0)	
	45–54 years	2 (14.3)	3 (11.5)	
	55–64 years	5 (35.7)	14 (53.8)	
	>65 years	0 (0)	9 (34.6)	
Ethnicity	Hispanic	2 (14.3)	6 (23.1)	0.507
	Non-Hispanic	12 (85.7)	20 (76.9)	
Race	White	9 (64.3)	9 (34.6)	0.072
	Non-White	5 (35.7)	17 (65.4)	
Status	Prediabetes	0 (0)	23 (88.5)	**<0.001**
	T2D	0 (0)	3 (11.5)	

Participant characteristics. Gender, age, diabetes and medication status clinically confirmed. Race and ethnicity information obtained through self-report. Significance shown in bold.

**Table 2:** Descriptive characteristics.

Variable	N-GLYC (n = 14)	D-GLYC (n = 26)	*p* Value
Anthropometrics			
Systolic BP	113.8 ± 10.9	121.9 ± 12.8	0.052
Diastolic BP	71.0 ± 9.2	77.9 ± 9.8	0.036 *
Height (cm)	170.2 ± 10.6	165.4 ± 9.8	0.154
Weight (kg)	71.5 ± 11.8	88.9 ± 19.3	0.004 *
BMI (kg/m^2^)	24.9 ± 5.1	32.3 ± 5.3	<0.001 *
Glucose			
Glycosylated hemoglobin (HbA1c)	N/A	5.9 ± 0.3	N/A
Homeostatic model assessment for insulin resistance (HOMA-IR)	N/A	4.9 ± 4.1	N/A
Total number of CGM readings	1117 ± 277	962 ± 201	<0.001 *
Mean glucose	96.40 ± 10.37	97.5 ± 10.4	0.754
Glucose variability	18.6 ± 4.7	19.7 ± 7.7	0.865
Mean amplitude of glycemic excursions (MAGE)	46.5 ± 10.8	51.8 ± 20.7	0.610
Largest amplitude of glycemic excursions (LAGE)	130.93 ± 23.61	136.8 ± 44.3	0.585
Min glucose reading	50.1 ± 11.9	52.2 ± 9.8	0.496
Max glucose reading	181.0 ± 21.7	189.0 ± 42.5	1.000
% in range	93.0 ± 7.9	94.8 ± 6.4	0.744
Eating patterns			
Number of days logging	13.4 ± 1.9	11.1 ± 0.3	<0.001 *
Good logging days (%)	92.3 ± 12.7	91.3 ± 11.1	0.429
Daily number of EOs	6 ± 2.3	6.2 ± 2.8	0.966
Eating window duration (hh:mm)	13:37 ± 1:05	14:21 ± 2:05	0.452
Time of first eating occasion (hh:mm)	9:19 ± 1:51	9:15 ± 1:44	0.903
Time of last eating occasion (hh:mm)	20:09 ± 1:37	19:46 ± 1:51	0.522
Eating midpoint (hh:mm)	15:10 ± 1:51	14:40 ± 1:44	0.398
Dietary composition			
Mean number of recalls	5.4 ± 1.3	4.8 ± 1.0	0.050
Calories	1827 ± 415	1824.9 ± 628	0.571
Protein (gr)	81.5 ± 25.3	75.4 ± 19.89	0.403
Total fat (gr)	73.3 ± 24.0	82.7 ± 32.9	0.411
Carbohydrate (gr)	206.0 ± 47.5	189.4 ± 87.3	0.119
Sugar (gr)	81.7 ± 24.7	78.5 ± 39.6	0.379
Sugar-to-carbohydrate ratio	0.4 ± 0.1	0.4 ± 0.1	0.568
Fiber (gr)	20.0 ± 7.6	20.9 ± 11.6	0.989
Fiber-to-carbohydrate ratio	0.1 ± 0.0	0.1 ± 0.0	0.357
Protein (%)	17.9 ± 3.7	17.4 ± 3.9	0.496
Total fat (%)	35.4 ± 5.4	39.7 ± 7.0	0.051
Carbohydrate (%)	46.0 ± 7.2	43.3 ± 8.1	0.303
Alcohol (gr)	7.2 ± 5.9	4.6 ± 10.2	0.008 *

Nonparametric data compared using Mann–Whitney U test, parametric data compared with Student’s *t* test. * Significance shown in bold. Glucose variability defined as glucose SD. Abbreviations: CGM—continuous glucose monitor; EO—eating occasion; hh:mm—hours and minutes. N/A = data not available and group comparisons cannot be performed.

## Data Availability

The data presented in this study are available upon reasonable request from the corresponding author.

## Supplementary Materials

The following supporting information can be downloaded at: https://www.mdpi.com/article/10.3390/nu16091295/s1, Figure S1: Study timeline; Figure S2: Study flowchart. Table S1: Inclusion and exclusion criteria in both cohorts; Table S2: Body composition as predictor of glucose parameters by continuous glucose monitor, adjusted for sex. Table S3: Correlations between HOMA-IR and glucose parameters by continuous glucose monitor. Table S4: Correlations between eating pattern and diet composition with HOMA-IR.

## References

[1] Sheard N.F., Clark N.G., Brand-Miller J.C., Franz M.J., Pi-Sunyer F.X., Mayer-Davis E., Kulkarni K., Geil P. (2004). Dietary carbohydrate (amount and type) in the prevention and management of diabetes: A statement by the american diabetes association. Diabetes Care.

[2] Franz M.J., Bantle J.P., Beebe C.A., Brunzell J.D., Chiasson J.L., Garg A., Holzmeister L.A., Hoogwerf B., Mayer-Davis E., Mooradian A.D. (2002). Evidence-based nutrition principles and recommendations for the treatment and prevention of diabetes and related complications. Diabetes Care.

[3] Hall K.D., Ayuketah A., Brychta R., Cai H., Cassimatis T., Chen K.Y., Chung S.T., Costa E., Courville A., Darcey V. (2019). Ultra-Processed Diets Cause Excess Calorie Intake and Weight Gain: An Inpatient Randomized Controlled Trial of Ad Libitum Food Intake. Cell Metab..

[4] Mansoor N., Vinknes K.J., Veierød M.B., Retterstøl K. (2016). Effects of low-carbohydrate diets v. low-fat diets on body weight and cardiovascular risk factors: A meta-analysis of randomised controlled trials. Br. J. Nutr..

[5] Tobias D.K., Chen M., Manson J.E., Ludwig D.S., Willett W., Hu F.B. (2015). Effect of low-fat diet interventions versus other diet interventions on long-term weight change in adults: A systematic review and meta-analysis. Lancet Diabetes Endocrinol..

[6] Zimmet P.Z. (2017). Diabetes and its drivers: The largest epidemic in human history?. Clin. Diabetes Endocrinol..

[7] Blüher M. (2019). Obesity: Global epidemiology and pathogenesis. Nat. Rev. Endocrinol..

[8] Hutchison A.T., Regmi P., Manoogian E.N.C., Fleischer J.G., Wittert G.A., Panda S., Heilbronn L.K. (2019). Time-Restricted Feeding Improves Glucose Tolerance in Men at Risk for Type 2 Diabetes: A Randomized Crossover Trial. Obesity.

[9] Xiao Q., Garaulet M., Scheer F. (2019). Meal timing and obesity: Interactions with macronutrient intake and chronotype. Int. J. Obes..

[10] Almoosawi S., Vingeliene S., Karagounis L.G., Pot G.K. (2016). Chrono-nutrition: A review of current evidence from observational studies on global trends in time-of-day of energy intake and its association with obesity. Proc. Nutr. Soc..

[11] Palomar-Cros A., Srour B., Andreeva V.A., Fezeu L.K., Bellicha A., Kesse-Guyot E., Hercberg S., Romaguera D., Kogevinas M., Touvier M. (2023). Associations of meal timing, number of eating occasions and night-time fasting duration with incidence of type 2 diabetes in the NutriNet-Santé cohort. Int. J. Epidemiol..

[12] Frontoni S., Di Bartolo P., Avogaro A., Bosi E., Paolisso G., Ceriello A. (2013). Glucose variability: An emerging target for the treatment of diabetes mellitus. Diabetes Res. Clin. Pract..

[13] Dimova R., Chakarova N., Del Prato S., Tankova T. (2023). The Relationship Between Dietary Patterns and Glycemic Variability in People with Impaired Glucose Tolerance. J. Nutr..

[14] Prasad M., Fine K., Gee A., Nair N., Popp C.J., Cheng B., Manoogian E.N.C., Panda S., Laferrère B. (2021). A Smartphone Intervention to Promote Time Restricted Eating Reduces Body Weight and Blood Pressure in Adults with Overweight and Obesity: A Pilot Study. Nutrients.

[15] Martin C.K., Han H., Coulon S.M., Allen H.R., Champagne C.M., Anton S.D. (2009). A novel method to remotely measure food intake of free-living individuals in real time: The remote food photography method. Br. J. Nutr..

[16] Gill S., Panda S. (2015). A Smartphone App Reveals Erratic Diurnal Eating Patterns in Humans that Can Be Modulated for Health Benefits. Cell Metab..

[17] Kim J., Campbell A.S., de Ávila B.E., Wang J. (2019). Wearable biosensors for healthcare monitoring. Nat. Biotechnol..

[18] Santos-Báez L.S., Garbarini A., Shaw D., Cheng B., Popp C.J., Manoogian E.N.C., Panda S., Laferrère B. (2022). Time-restricted eating to improve cardiometabolic health: The New York Time-Restricted EATing randomized clinical trial—Protocol overview. Contemp. Clin. Trials.

[19] Blum A. (2018). Freestyle Libre Glucose Monitoring System. Clin. Diabetes.

[20] Hill N.R., Oliver N.S., Choudhary P., Levy J.C., Hindmarsh P., Matthews D.R. (2011). Normal reference range for mean tissue glucose and glycemic variability derived from continuous glucose monitoring for subjects without diabetes in different ethnic groups. Diabetes Technol. Ther..

[21] Subar A.F., Kirkpatrick S.I., Mittl B., Zimmerman T.P., Thompson F.E., Bingley C., Willis G., Islam N.G., Baranowski T., McNutt S. (2012). The Automated Self-Administered 24-hour Dietary Recall (ASA24): A resource for researchers, clinicians and educators from the National Cancer Institute. J. Acad. Nutr. Diet..

[22] Manoogian E.N.C., Wei-Shatzel J., Panda S. (2022). Assessing temporal eating pattern in free living humans through the myCircadianClock app. Int. J. Obes..

[23] Wilkinson M.J., Manoogian E.N.C., Zadourian A., Lo H., Fakhouri S., Shoghi A., Wang X., Fleischer J.G., Navlakha S., Panda S. (2020). Ten-Hour Time-Restricted Eating Reduces Weight, Blood Pressure, and Atherogenic Lipids in Patients with Metabolic Syndrome. Cell Metab..

[24] (2024). 2. Diagnosis and Classification of Diabetes: Standards of Care in Diabetes-2024. Diabetes Care.

[25] Sierra-Johnson J., Undén A.L., Linestrand M., Rosell M., Sjogren P., Kolak M., De Faire U., Fisher R.M., Hellénius M.L. (2008). Eating meals irregularly: A novel environmental risk factor for the metabolic syndrome. Obesity.

[26] Bernardes da Cunha N., Teixeira G.P., Madalena Rinaldi A.E., Azeredo C.M., Crispim C.A. (2023). Late meal intake is associated with abdominal obesity and metabolic disorders related to metabolic syndrome: A chrononutrition approach using data from NHANES 2015–2018. Clin. Nutr..

[27] Parr E.B., Steventon-Lorenzen N., Johnston R., Maniar N., Devlin B.L., Lim K.H.C., Hawley J.A. (2023). Time-restricted eating improves measures of daily glycaemic control in people with type 2 diabetes. Diabetes Res. Clin. Pract..

[28] Parr E.B., Devlin B.L., Radford B.E., Hawley J.A. (2020). A Delayed Morning and Earlier Evening Time-Restricted Feeding Protocol for Improving Glycemic Control and Dietary Adherence in Men with Overweight/Obesity: A Randomized Controlled Trial. Nutrients.

[29] Chow L.S., Manoogian E.N.C., Alvear A., Fleischer J.G., Thor H., Dietsche K., Wang Q., Hodges J.S., Esch N., Malaeb S. (2020). Time-Restricted Eating Effects on Body Composition and Metabolic Measures in Humans who are Overweight: A Feasibility Study. Obesity.

[30] Samkani A., Skytte M.J., Thomsen M.N., Astrup A., Deacon C.F., Holst J.J., Madsbad S., Rehfeld J.F., Krarup T., Haugaard S.B. (2018). Acute Effects of Dietary Carbohydrate Restriction on Glycemia, Lipemia and Appetite Regulating Hormones in Normal-Weight to Obese Subjects. Nutrients.

[31] Samkani A., Skytte M.J., Kandel D., Kjaer S., Astrup A., Deacon C.F., Holst J.J., Madsbad S., Rehfeld J.F., Haugaard S.B. (2018). A carbohydrate-reduced high-protein diet acutely decreases postprandial and diurnal glucose excursions in type 2 diabetes patients. Br. J. Nutr..

[32] Stephenson E.J., Smiles W., Hawley J.A. (2014). The Relationship between Exercise, Nutrition and Type 2 Diabetes.

[33] Gannon M.C., Nuttall F.Q. (2004). Effect of a high-protein, low-carbohydrate diet on blood glucose control in people with type 2 diabetes. Diabetes.

[34] Basu S., Yoffe P., Hills N., Lustig R.H. (2013). The relationship of sugar to population-level diabetes prevalence: An econometric analysis of repeated cross-sectional data. PLoS ONE.

[35] Dorans K.S., Bazzano L.A., Qi L., He H., Chen J., Appel L.J., Chen C.S., Hsieh M.H., Hu F.B., Mills K.T. (2022). Effects of a Low-Carbohydrate Dietary Intervention on Hemoglobin A1c: A Randomized Clinical Trial. JAMA Netw. Open.

[36] Wolever T.M., Nuttall F.Q., Lee R., Wong G.S., Josse R.G., Csima A., Jenkins D.J. (1985). Prediction of the relative blood glucose response of mixed meals using the white bread glycemic index. Diabetes Care.

[37] Wolever T.M., Miller J.B. (1995). Sugars and blood glucose control. Am. J. Clin. Nutr..

[38] Huang T., Xu M., Lee A., Cho S., Qi L. (2015). Consumption of whole grains and cereal fiber and total and cause-specific mortality: Prospective analysis of 367,442 individuals. BMC Med..

[39] Oba-Yamamoto C., Takeuchi J., Nakamura A., Nomoto H., Kameda H., Cho K.Y., Atsumi T., Miyoshi H. (2022). Impact of low-starch high-fiber pasta on postprandial blood glucose. Nutr. Metab. Cardiovasc. Dis..

[40] Korczak R., Slavin J.L. (2020). Definitions, regulations, and new frontiers for dietary fiber and whole grains. Nutr. Rev..

[41] Tettamanzi F., Bagnardi V., Louca P., Nogal A., Monti G.S., Mambrini S.P., Lucchetti E., Maestrini S., Mazza S., Rodriguez-Mateos A. (2021). A High Protein Diet Is More Effective in Improving Insulin Resistance and Glycemic Variability Compared to a Mediterranean Diet-A Cross-Over Controlled Inpatient Dietary Study. Nutrients.

[42] Goyenechea E., Holst C., van Baak M.A., Saris W.H., Jebb S., Kafatos A., Pfeiffer A., Handjiev S., Hlavaty P., Stender S. (2011). Effects of different protein content and glycaemic index of ad libitum diets on diabetes risk factors in overweight adults: The DIOGenes multicentre, randomized, dietary intervention trial. Diabetes Metab. Res. Rev..

[43] de la Cruz-Ares S., Gutiérrez-Mariscal F.M., Alcalá-Díaz J.F., Quintana-Navarro G.M., Podadera-Herreros A., Cardelo M.P., Torres-Peña J.D., Arenas-de Larriva A.P., Pérez-Martínez P., Delgado-Lista J. (2021). Quality and Quantity of Protein Intake Influence Incidence of Type 2 Diabetes Mellitus in Coronary Heart Disease Patients: From the CORDIOPREV Study. Nutrients.

[44] Fappi A., Mittendorfer B. (2020). Dietary protein intake and obesity-associated cardiometabolic function. Curr. Opin. Clin. Nutr. Metab. Care.

[45] Chan K.K., Neighbors C., Gilson M., Larimer M.E., Alan Marlatt G. (2007). Epidemiological trends in drinking by age and gender: Providing normative feedback to adults. Addict. Behav..

[46] Tarragon E. (2023). Alcohol and energy drinks: Individual contribution of common ingredients on ethanol-induced behaviour. Front. Behav. Neurosci..

[47] Bantle A.E., Thomas W., Bantle J.P. (2008). Metabolic effects of alcohol in the form of wine in persons with type 2 diabetes mellitus. Metabolism.

[48] Ma Y., Olendzki B.C., Pagoto S.L., Hurley T.G., Magner R.P., Ockene I.S., Schneider K.L., Merriam P.A., Hébert J.R. (2009). Number of 24-hour diet recalls needed to estimate energy intake. Ann. Epidemiol..

[49] Park J.H., Lee M.Y., Shin H.K., Yoon K.J., Lee J., Park J.H. (2023). Lower skeletal muscle mass is associated with diabetes and insulin resistance: A cross-sectional study. Diabetes Metab. Res. Rev..

[50] Mizgier M.L., Casas M., Contreras-Ferrat A., Llanos P., Galgani J.E. (2014). Potential role of skeletal muscle glucose metabolism on the regulation of insulin secretion. Obes. Rev..

[51] Wyatt P., Berry S.E., Finlayson G., O’Driscoll R., Hadjigeorgiou G., Drew D.A., Khatib H.A., Nguyen L.H., Linenberg I., Chan A.T. (2021). Postprandial glycaemic dips predict appetite and energy intake in healthy individuals. Nat. Metab..

