# Supplementary material for: Diet and Meal Pattern Determinants of Glucose Levels and Variability in Adults with and without Prediabetes or Early-Onset Type 2 Diabetes: A Pilot Study
Source: Nutrients. 2024 Apr 26;16(9):1295. doi: 10.3390/nu16091295 (PMC11085124; doi:10.3390/nu16091295)
Supplement: Supplementary file 1 [file nutrients-16-01295-s001.zip › nutrients-2921126-supplementary.pdf]

Contents

Figure S1. Study timeline 1

Figure S2. Study flowchart 2

Table S1. Inclusion and exclusion criteria in both cohorts. 3

Table S2. Body composition as predictor of glucose parameters by continuous glucose monitor, adjusted for sex. 4

Table S3. Correlations between HOMA-IR and glucose parameters by continuous glucose monitor. 5

Table S4. Correlations between eating pattern and diet composition with HOMA-IR. 6

Figure S1. Study timeline

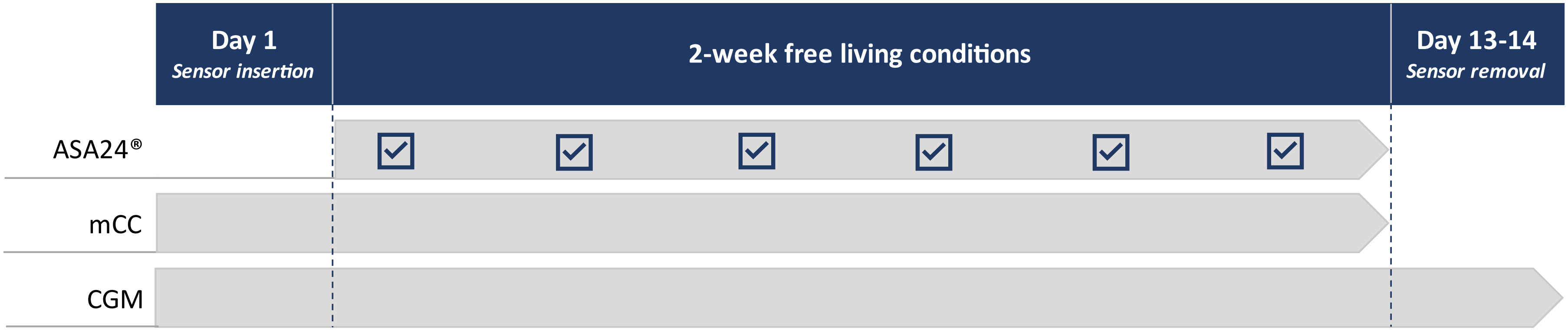

Study procedures timeline in both groups. Sensor insertion is defined as day of continuous glucose monitor (CGM) insertion, for N-GLYC, the CGM glucose data were used from day 2 to 14, in D-GLYC, CGM data only included days 2 to 12, as the diet was controlled on days 13 and 14. The Automated Self-Administered 24-hour® (ASA24®) were completed by each participant on non-consecutive weekdays and at least one weekend day over 2-week period, for a total of 3 to 6 recalls; the myCircadianClock phone application (mCC) was used daily by each participant to record time-stamped photos, in real time, of all food and caloric beverages immediately prior to ingestion.

Figure S2. Study flowchart

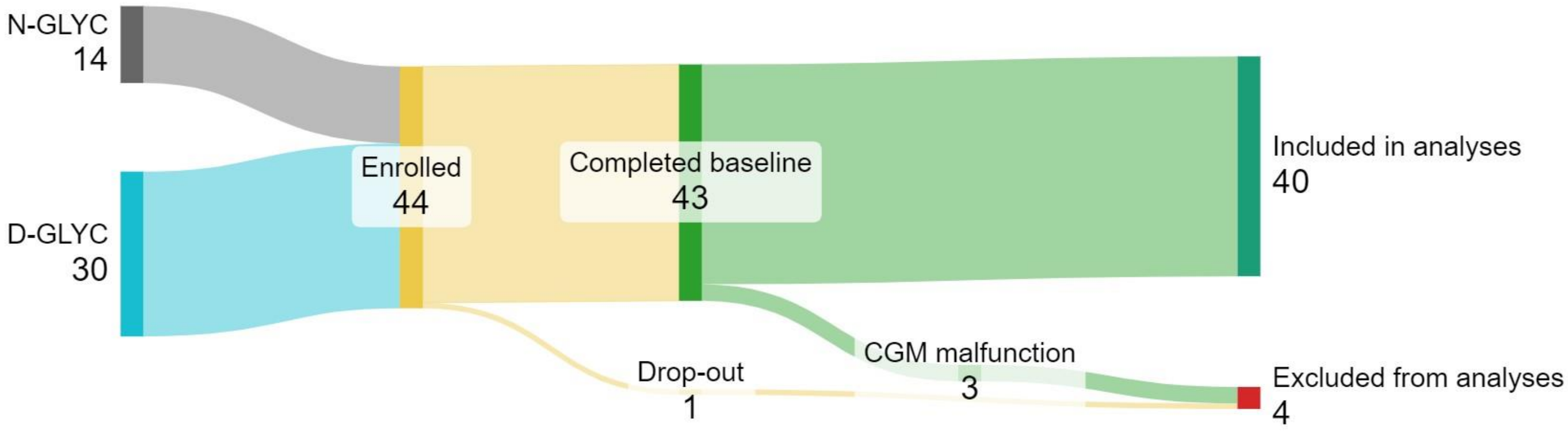

Participant selection process for N-GLYC and D-GLYC study analyses after consent. Abbreviations: CGM = continuous glucose monitor.

Table S1. Inclusion and exclusion criteria in both cohorts.

N-GLYC	D-GLYC
Inclusion criteria	
Age: > 18 years old	Age: 50-75 years old
BMI >20, but less than <35, with weight stability within 5% for the 3 months preceding screening	BMI ≥25 and ≤45kg/m^2, with weight stability within 5% for the 3 months preceding screening
Self-reported fasting glucose <100 mg/dL, and/or HbA1c <5.7%<br><br>No history of prediabetes or type 2 diabetes (T2D), and absence of any glucose-lowering agents prescriptions	History of prediabetes or T2D<br><br>Fasting glucose ≥100 mg/dL, and/or HbA1c ≥5.7% and <7.5%<br><br>Treatment with metformin and/or diet-controlled if T2D
	Habitual prolonged eating window (≥14 hours)
Habitually eats breakfast	Habitually eats breakfast
No medical history of metabolic syndrome	Two or more of the following metabolic syndrome criteria:<br>- Diagnosis of hypertension on stable medication regimen<br>- Diagnosis of dyslipidemia on stable regimen<br>- HDL cholesterol men <40 mg/dL and women <50 mg/dL<br>- Waist circumference men: >102 cm (>40 40 in); women >88 cm (>35 in)
Sleep duration ≥6 hours, with habitual self-reported wake-up time >5:00 am and <11:00 am, and average self-reported bedtime before 2:00 am	Sleep duration ≥6 hours, with habitual self-reported wake-up time >5:00 am and <11:00 am, and average self-reported bedtime before 2:00 am
	≥70% of days with logging adherence (2 or more log entries/day separated by at least 5h), assessed during 2-week remote screening
In possession of a smartphone	In possession of a smartphone
Lives in the New York City metro area	Lives in the New York City metro area
English fluency, since the smartphone application has not been translated	English fluency, since the smartphone application has not been translated
Exclusion criteria	
Sleep disorder, e.g. known obstructive sleep apnea (OSA), severe self-reported diagnosis of OSA, significant daytime symptoms of OSA, periodic limb movements of sleep, narcolepsy, or severe insomnia<br><br>Current shift work or recent shift work in the last 6 months<br><br>Travel during the observational period	Sleep disorder, e.g. known obstructive sleep apnea (OSA), severe OSA with apnea-hypopnea index >30 events/hour, significant daytime symptoms of OSA, periodic limb movements of sleep, narcolepsy, severe insomnia (a score ≥15 on Insomnia Severity Index)<br><br>Current shift work or recent shift work in the last 6 months<br><br>Travel more than one time zone during the intervention
Previous history of bariatric surgery or use of weight loss medication	Previous history of bariatric surgery or use of weight loss medication
Use of dietary supplements and/or medications known to affect sleep, circadian rhythms, or metabolic function<br><br>Self-reported history of or current significant food intake or psychiatric disorder<br><br>Excessive alcohol (women: >14 drinks/week; men: >21 drinks/week), smoking tobacco, or using illegal or recreational drugs	Use of dietary supplements and/or medications known to affect sleep, circadian rhythms, or metabolic function<br><br>History of or current significant food intake or psychiatric disorder (BDI score ≥29; BAI score ≥26)<br><br>Excessive alcohol (women: >14 drinks/week; men: >21 drinks/week), smoking tobacco, or using illegal or recreational drugs
History of seizure disorder	History of seizure disorder
Unwilling/unable to provide informed consent	Unwilling/unable to provide informed consent
	Severe food allergies

Abbreviations: BMI = Body Mass Index; HbA1c = glycated hemoglobin; HDL = High-Density Lipoprotein; OSA = Obstructive Sleep Apnea; T2D = Type 2 Diabetes.

Table S2. Body composition as predictor of glucose parameters by continuous glucose monitor, adjusted for sex.

Predictor	FM (%)			FFM (%)		
	B	SE	Sig.	B	SE	p value
Mean glucose	-0.282	0.379	0.464	0.282	0.379	0.464
Glucose variability	-0.018	0.283	0.950	0.018	0.283	0.950
MAGE	-0.004	0.769	0.996	0.004	0.769	0.996
LAGE	-0.098	1.645	0.953	0.098	1.645	0.953

Generalized linear model between body composition and glucose parameters by continuous glucose monitor (CGM) in the D-GLYC group (n = 26). Fat mass (FM) was assessed via quantitative magnetic resonance (QMR) under fasting condition in duplicate[16]; Fat-free mass (FFM) was calculated by subtracting FM from body weight. The percentage of FM was calculated as the total FM (kg) by QMR divided by total body weight; the percentage of FFM was calculated as the total FFM (kg) divided by total body weight. FM and FFM associations were adjusted for sex, to eliminate biological confounders from differences in FM/FFM distribution between sexes. Significance set at $p < 0.05$. Glucose variability defined as glucose SD.
Abbreviations: FFM = fat-free mass; FM= fat mass LAGE = largest amplitude of glycemic excursions; MAGE = mean amplitude of glycemic excursions.

Table S3. Correlations between HOMA-IR and glucose parameters by continuous glucose monitor.

Glucose parameter	HOMA-IR	
	Coefficient	p value
Mean glucose	0.073	0.724
Glucose variability	-0.245	0.227
MAGE	-0.240	0.237
LAGE	-0.345	0.084

Correlations between HOMA-IR and glucose parameters by continuous glucose monitor in the D-GLYC group (n = 26). Glucose variability defined as glucose SD. Significance set at $p < 0.05$. Abbreviations: HOMA-IR = Homeostatic Model Assessment for Insulin Resistance; LAGE = largest amplitude of glucose excursions; MAGE = mean amplitude of glucose excursions.

Table S4. Correlations between eating pattern and diet composition with HOMA-IR.

Predictors	Variable	HOMA-IR	
		Coefficient	p value
Eating pattern	Daily EO	-0.04	0.84
	Eating window, hrs	0.23	0.25
	First EO	0.04	0.86
	Last EO	0.17	0.40
	Eating midpoint	0.17	0.42
Diet composition	Calories	-0.27	0.18
	CHO, gr	-0.38	0.06
	CHO, %	-0.22	0.27
	Fiber	**-0.44**	**0.03**
	Fiber-to-CHO	-0.32	0.12
	Sugar	-0.32	0.11
	Sugar-to-CHO	0.21	0.31
	Protein, gr	-0.08	0.68
	Protein, %	0.17	0.40
	Total fat, gr	-0.12	0.57
	Total fat, %	0.21	0.29
	ETOH, gr	**-0.41**	**0.04**

Predictors	Variable	HOMA-IR	
		Coefficient	p value
Eating pattern	Daily EO, SD	0.02	0.94
	First EO, SD	0.04	0.85
	Last EO, SD	0.09	0.66
	Eating midpoint, SD	0.07	0.75
Diet composition	Calories, SD	-0.05	0.80
	CHO, gr, SD	-0.30	0.14
	CHO, %, SD	-0.08	0.70
	Fiber, SD	**-0.53**	**0.01**
	Fiber-to-CHO, SD	-0.37	0.06
	Sugar, SD	-0.23	0.25
	Sugar-to-CHO, SD	-0.17	0.41
	Protein, gr, SD	-0.14	0.50
	Protein, %, SD	0.07	0.75
	Total fat, gr, SD	0.05	0.80
	Total fat, %, SD	-0.07	0.74
	ETOH, gr, SD	-0.36	0.07

Correlations between eating patterns and diet composition with HOMA-IR in the D-GLYC group (n = 26). Left, eating pattern and dietary composition over two weeks correlations with HOMA-IR. Right, eating pattern and dietary composition variability over two weeks correlations with HOMA-IR. Significance shown in bold. Abbreviations: CHO = carbohydrate; EO = eating occasion; ETOH = alcohol; HOMA-IR = Homeostatic Model Assessment for Insulin Resistance.
